# Comparative Genomic and Transcriptomic Analysis of Phenol Degradation and Tolerance in *Acinetobacter lwoffii* through Adaptive Evolution

**DOI:** 10.3390/ijms242216529

**Published:** 2023-11-20

**Authors:** Nan Xu, Xiaojing Yang, Qiyuan Yang, Minliang Guo

**Affiliations:** College of Bioscience and Biotechnology, Yangzhou University, Yangzhou 225009, China

**Keywords:** *Acinetobacter lwoffii*, phenol, bioremediation, alkyl hydroperoxide reductase

## Abstract

Microorganism-based methods have been widely applied for the treatment of phenol-polluted environments. The previously isolated *Acinetobacter lwoffii* NL1 strain could completely degrade 0.5 g/L phenol within 12 h, but not higher concentrations of phenol. In this study, we developed an evolutionary strain NL115, through adaptive laboratory evolution, which possessed improved degradation ability and was able to degrade 1.5 g/L phenol within 12 h. Compared with that of the starting strain NL1, the concentration of degradable phenol by the developed strain increased three-fold; its phenol tolerance was also enhanced. Furthermore, comparative genomics showed that sense mutations mainly occurred in genes encoding alkyl hydroperoxide reductase, phenol hydroxylase, 30S ribosomal protein, and mercury resistance operon. Comparative transcriptomics between *A. lwoffii* NL115 and NL1 revealed the enrichment of direct degradation, stress resistance, and vital activity processes among the metabolic responses of *A. lwoffii* adapted to phenol stress. Among these, all the upregulated genes (log_2_fold-change > 5) encoded peroxidases. A phenotypic comparison of *A. lwoffii* NL1 and NL115 found that the adapted strain NL115 exhibited strengthened antioxidant capacity. Furthermore, the increased enzymatic activities of phenol hydroxylase and alkyl hydroperoxide reductase in *A. lwoffii* NL115 validated their response to phenol. Overall, this study provides insight into the mechanism of efficient phenol degradation through adaptive microbial evolution and can help to drive improvements in phenol bioremediation.

## 1. Introduction

Phenol, a model compound of aromatic phenols, has been categorized as a hazardous pollutant associated with difficulties in natural degradation, and it is also easily converted into other deleterious aromatic compounds [1]. The microbial biodegradation of phenol has received increasing attention because of its environmental friendliness and relatively low degradation costs [2]. Studies on microbial degradation and tolerance to high phenol concentrations can also guide the biodegradation of other aromatic pollutants [3,4]. Microbial phenol degradation can be determined by the microbial type and quantity, pollutant concentration, and environmental conditions. Promising pure cultures that can degrade high phenol concentrations include the yeasts *Candida albicans* PDY-07 (1.8 g/L, 68 h) [5] and *Candida tropicalis* JH8 (1.8 g/L, 66 h) [6] and the bacteria *Acinetobacter johnsonii* D1 (1.35 g/L, 144 h) [7] and *Bacillus brevis* (1.5 g/L, 108 h) [8]. Further, the previously isolated *Acinetobacter lwoffii* NL1, with the highest efficiency, could degrade up to 0.5 g/L phenol within 12 h under the appropriate conditions (pH 7.0, 28 °C, and 2% inoculum) [9]. *A. lwoffii* NL1 had one circular chromosome and three plasmids in its genome. A megaplasmid (pNL1) contained genes encoding phenol hydroxylase (*LSNL_2975-2980*) and catechol 1,2-dioxygenase (*LSNL_2981*). Genome analysis indicated *A. lwoffii* NL1 had the potential for degrading other aromatic compounds such as benzoate and salicylate. However, *A. lwoffii* NL1 could not grow on a mineral medium containing more than 0.6 g/L of phenol as the sole carbon source, thus limiting its application in the bioremediation of wastewater contaminated with higher concentrations of phenol.

To improve degradation capacity, biotechnological approaches, including mutagenesis, adaptive evolution, genetic engineering, and the immobilization of microbial cells, have been commonly employed. Among these methods, adaptive laboratory evolution (ALE) is not dependent on the knowledge of complex metabolic mechanisms; therefore, the target microorganism can easily adapt to artificial selection stress. Microbial ALE has been used in biotechnological fields for the production of high-value products, biodegradation of toxic pollutants, and enhancement of microbial environmental adaptation [10,11]. The phenol degradation capacity of *Rhodococcus opacus* [12], *Rhodococcus pyridinivorans* [13], *Acinetobacter* sp. [14], *Chlorella* sp. [15], and *Is Chrysis galbana Parke* [16] has been improved using ALE. Moreover, adaptation to an evolutionary phenol environment is accompanied by changes in fitness and degradation capacity. The mechanisms underlying phenotypic changes occur across multiple layers of genetics and through transcriptional regulation and metabolism, which has commonly been explained through genomic and post-genomic analyses of the evolved mutants [17].

In this study, the previously isolated strain *A. lwoffii* NL1 was acclimated by continuously increasing the phenol concentration. Through approximately 80 cycles, strain NL115 completely degraded 1.5 g/L phenol in 12 h. ALE thus enhanced the tolerance and biodegradation of high-level phenol. To reveal the underlying alterations, we performed comparative genomic and transcriptomic analyses of *A. lwoffii* NL1 and NL115. This combined approach was effective in identifying differentially expressed genes (DEGs). Some of these genes and respective proteins were further studied for their gene expression, enzymatic activity, and biochemical phenotypes. This study uncovers a highly efficient phenol-degrading strain and explores the underlying mechanism of adaptive evolution in response to high-phenol stress and will be useful for metabolic engineering for microbial phenol degradation and tolerance.

## 2. Results

### 2.1. ALE under High-Phenol Stress

**Figure 1:**
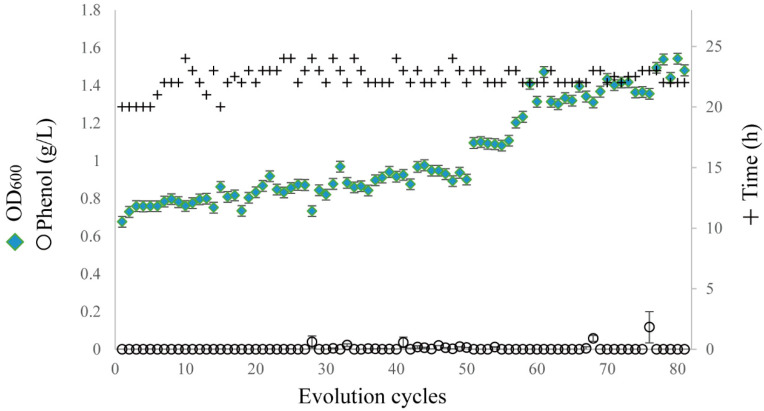
Adaptive evolution cycle of *Acinetobacter lwoffii* NL1. The duration of each cycle was denoted by + symbols, which reflected the time spent in each cycle. The final biomass (OD_600_) in each cycle of the adaptive evolution process was represented by green square symbols. The surplus phenol in each cycle was represented by the circle symbols.

*A. lwoffii* NL1 did not grow normally when the phenol concentration exceeded 0.6 g/L. To improve phenol degradability, ALE was performed in a liquid MM medium with an increasing concentration of phenol as the sole carbon source. Starting from 0.5 g/L as the initial phenol concentration, the phenol was increased by 10 mg/L in each cycle of evolution. If the time required for phenol degradation was shorter than that in the previous cycle, the evolutionary gradient could be increased by 20–50 mg/L in the next cycle. As shown in Figure 1, the final biomass in each cycle gradually increased with the number of evolutionary cycles, suggesting that these adapted strains grew well using phenol as the sole carbon source. After 80 serial transfers, the concentration of the degradable phenol was increased to 1.5 g/L. The resulting bacterial solution was diluted and seeded onto phenol-containing agar plates. A fast-growing single colony (marked as NL115) was tested in liquid MM with 1.5 g/L phenol as the sole carbon source. This strain, NL115, completely degraded 1.5 g/L phenol after 12 h, and the OD_600_ reached 1.652 at 12 h. The relationship between cell growth and phenol degradation generally conformed to a synchronous model.

### 2.2. Phenotypic Comparison between A. lwoffii NL1 and NL115

**Figure 2:**
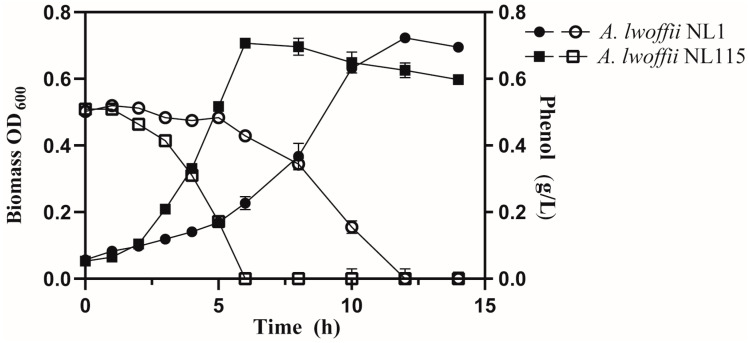
*Acinetobacter lwoffii* NL1 and NL115 grown on 0.5 g/L phenol-containing liquid minimal mineral medium. The solid symbols represent cell growth, and the hollow symbols represent phenol degradation. The circle and square symbols represent *A. lwoffii* NL1 and NL115, respectively.

*A. lwoffii* NL1 and NL115 were compared in 0.5 g/L phenol-containing liquid MM medium. As shown in Figure 2, compared to the starting strain NL1, *A. lwoffii* NL115 showed obvious growth advantages. When inoculated into an environment containing 0.5 g/L phenol, *A. lwoffii* NL115 rapidly entered its logarithmic period, and this strain required 6 h to reach its maximum biomass, whereas *A. lwoffii* NL1 required 12 h. In terms of phenol degradation, the average efficiency of *A. lwoffii* NL115 was 0.083 g/(L·h), which was almost twice that of *A. lwoffii* NL1. The good cell growth and phenol biodegradation of *A. lwoffii* NL115 were thus inseparable from its adaptation to high-phenol stress.

**Figure 3:**
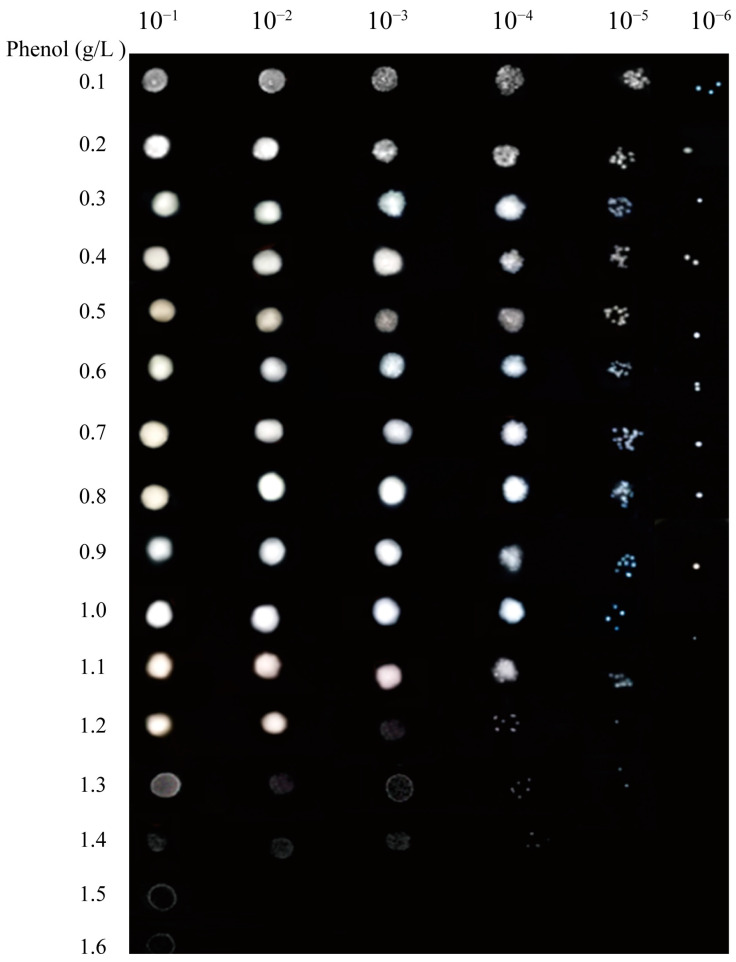
Pot assays of *Acinetobacter lwoffii* NL115 growth on plates with different phenol concentrations.

The phenol tolerance of *A. lwoffii* NL115 was then evaluated on MM plates containing phenol at concentrations ranging from 0 to 1.6 g/L. As shown in Figure 3, visible colonies were formed on 0.1–0.9 g/L phenol plates even at the maximum dilution of the bacterial concentration. On 1.0–1.3 g/L phenol plates, few colonies were formed at the 10^−5^ bacterial concentration. Cell growth was inhibited on 1.3–1.6 g/L phenol plates even at the 10^−1^ bacterial concentration. The maximum concentration tolerated by strain NL115 on MM plates was approximately 1.5 g/L, which was slightly higher than that tolerated by the original strain NL1 [9]. Cell growth of *A. lwoffii* NL115 was also examined on MM plates with sodium acetate serving as the carbon source. At varying concentrations, the C molar masses of phenol and sodium acetate were identical. *A. lwoffii* NL115 was able to thrive on NaAc as a carbon source at all test doses, but it was unable to form a colony on phenol at concentrations greater than 1.3 g/L, according to a comparison of cell growth on phenol and NaAc plates at 10^−5^ diluted concentrations. (Figure S1, Supplementary File).

**Figure 4:**
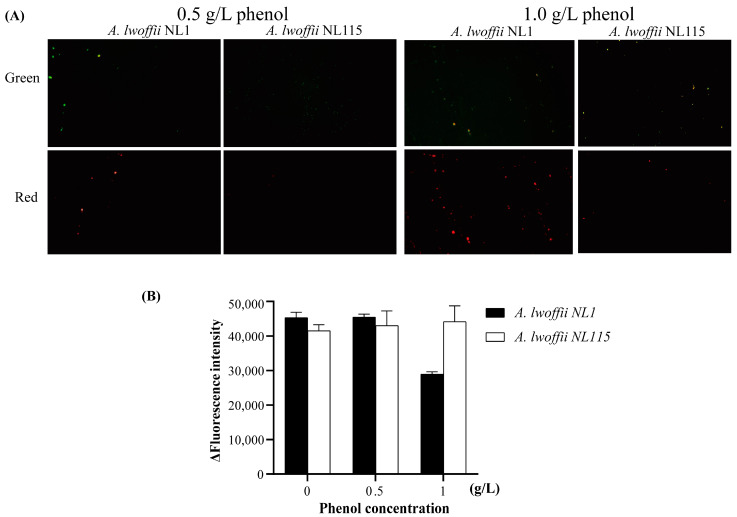
Effects of phenol on cell death. (**A**) Acridine orange/ethidium bromide (AO/EB) fluorescence staining of *A. lwoffii* NL1 and NL115 under phenol stress. Green indicates the use of green filters; Red indicates the use of red filters. (**B**) AO fluorescence intensity in *A. lwoffii* NL1 and NL115 at various phenol concentrations.

Fluorescence microscopy was used to qualitatively evaluate the survival of cells treated with phenol after acridine orange (AO) and ethidium bromide (EB) staining. Live cells will appear uniformly green, while early apoptotic cells will have condensed or fragmented nuclei with a bright green color. Late apoptotic cells will show condensed and fragmented orange chromatin [18]. Phenol treatment of *A. lwoffii* NL1 and NL115 will lead to partial cell damage due to its toxicity effect. These suggest that, following AO/EB staining, the color of the cells, as seen by fluorescence microscopy, may represent their vitality in some way. As shown in Figure 4A, under 0.5 g/L phenol stress, green cells were more abundant than red cells for *A. lwoffii* NL1, whereas many living cells, but no obvious cell death, were observed for *A. lwoffii* NL115. Red cell count rose as phenol content increased from 0.5 to 1.0 g/L. The relative ratio of green to red cells was higher for *A. lwoffii* NL115 than for *A. lwoffii* NL1. In addition, the survival of cells treated with phenol after acridine orange staining was quantified by measuring fluorescence intensity with a Microplate reader. Acridine orange can cross normal cell membranes. After AO staining, the nuclei of normal cells exhibit green or yellow–green fluorescence. Dead cells’ fluorescence decreases or even disappears. As shown in Figure 4B, the fluorescence intensity of *A. lwoffii* NL1 and NL115 was nearly the same after 4 h of treatment with 0.5 g/L phenol. The fluorescence intensity of *A. lwoffii* NL1 was 64% of the untreated group after 4 h of 1.0 g/L phenol treatment, whereas the fluorescence intensity of *A. lwoffii* NL115 was 1.06 times of the untreated group. The quantitative data indicated that *A. lwoffii* NL1 died, whereas *A. lwoffii* NL115 survived 1.0 g/L phenol stress. Both the qualitative investigation and quantitative comparison confirmed the adapted strain NL115 had a higher tolerance.

### 2.3. Comparative Genomics and Transcriptomics of A. lwoffii NL1 and NL115

The genome of the adapted strain NL115 was sequenced using the *A. lwoffii* NL1 reference genome sequence. Here, 1748 scaffolds and 2634 contigs were assembled with a read depth of 212×. The coverage rate mapped to the reference genome was 99.95%. The 192 single-nucleotide polymorphisms in the gene-coding regions included 144 synonymous and 48 non-synonymous mutations (Table S1). Sense mutations were found in genes related to the mercury resistance operon (*LNSL_3430*, *3433–3435)* on the plasmid pNL3, *LNSL_1974* (alkyl hydroperoxide reductase F subunit) on chromosome, *LSNL_2510* (30S ribosomal protein S10) on chromosome, and *LNSL_2977* (phenol hydroxylase L subunit) on the megaplasmid pNL1. Compared to the sequences of respective genes in *A. lwoffii* NL1, very few indels were distributed in *LNSL_3434* (transposase), *LNSL_0851* (alpha-ketoglutarate dehydrogenase E1 subunit), and *LNSL_3492* sequences of NL115.

**Figure 5:**
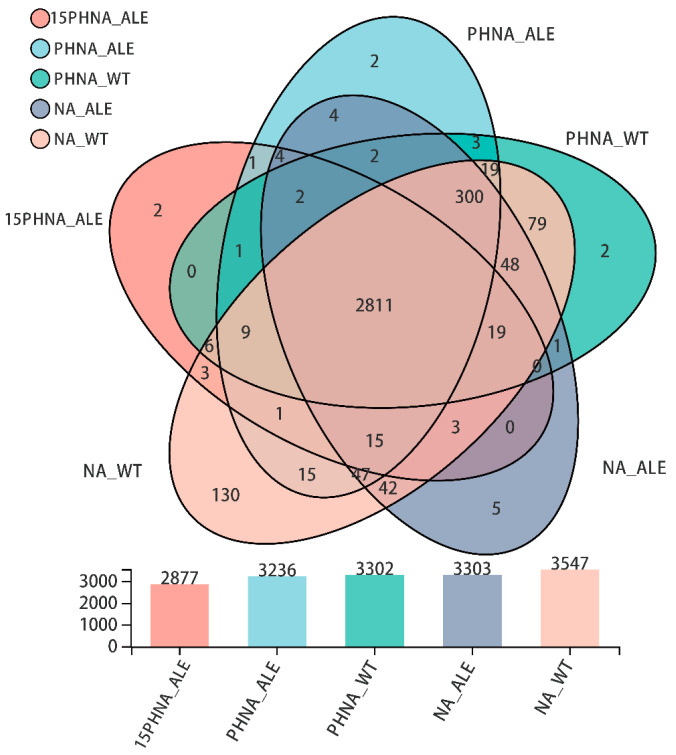
Venn diagrams for RNA-seq discovered genes in each of the five conditions.

RNA sequencing of *A. lwoffii* NL1 and NL115 during mid-exponential growth was performed in three different media. As shown in Figure 5, in the NaAc medium, 3303 and 3547 expressed genes were detected in *A. lwoffii* NL115 and NL1, respectively. On NaAc medium with the addition of 0.5 g/L phenol, 3302 and 3303 genes were expressed in *A. lwoffii* NL115 and NL1, respectively. In the NaAc medium with the addition of 1.5 g/L phenol, 2877 expressed genes were detected in *A. lwoffii* NL115. To validate the accuracy of the RNA sequencing, 15 DEGs in Table S2 were selected for qRT-PCR analysis, and their expression levels are presented in Figure S2. Table S3 lists these qPCR primers, and the melting curves of the qPCR products are shown in Figure S3. The correlation coefficient (R^2^ = 0.8533) showed that the RNA-Seq data reflected the actual gene expression trends (Figure S4).

15PHNA_ALE: *A. lwoffii* NL115 was cultivated on the medium containing 1.3 g/L NaAc and 1.5 g/L phenol. PHNA_ALE and PHNA_WT: *A. lwoffii* NL115 and *A. lwoffii* NL1 were cultivated on the medium containing 1.3 g/L NaAc and 0.5 g/L phenol, respectively. NA_ALE and NA_WT: *A. lwoffii* NL115 and *A. lwoffii* NL1 were cultivated on 1.3 g/L NaAc medium.

**Figure 6:**
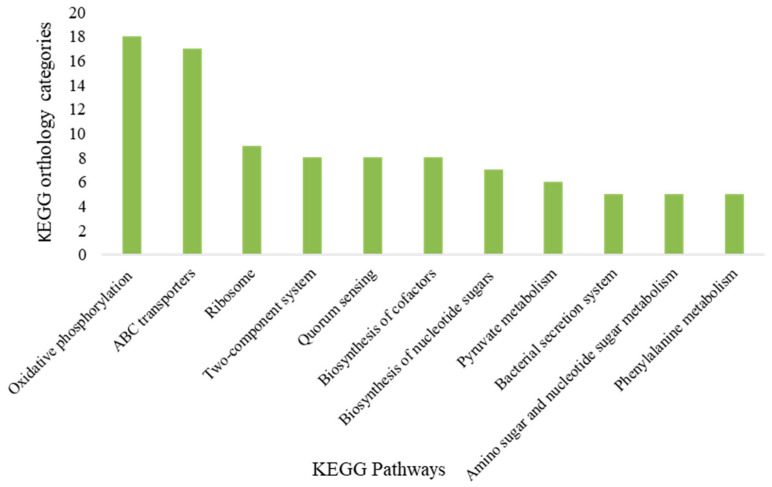
Enriched downregulated metabolic pathways in *Acinetobacter lwoffii* NL1 and NL115 under phenol stress. The ordinate corresponds to the number of KO categories in each metabolic pathway.

The transcriptome profiles of *A. lwoffii* NL115 and NL1 in the 0.5 g/L phenol-containing medium were compared to those in the NaAc medium. The DEGs shared by the two strains included 61 upregulated and 435 downregulated genes (Table S4). Functional annotation of the upregulated DEGs revealed that they were primarily related to phenol degradation. In aerobic bacteria, phenol degradation is mostly dependent on ortho- or meta-cleavage. *A. lwoffii* NL1 degraded phenol via the ortho-cleavage, and key genes encoding phenol hydroxylase and catechol 1,2-dioxygenase were located on a megaplasmid (pNL1) [9]. In addition to the known ortho-cleavage gene cluster on the megaplasmid pNL1 (locus tags *LSNL_2975–2981*), the upregulated DEGs also included *LSNL_1566–1571* on the circular chromosome and *LNSL_3112* on pNL1. *LNSL_3112* was annotated as encoding the phenol hydroxylase P_0_ protein, and *LSNL_1566–1568* was annotated as being involved in the first three steps of the catechol ortho-cleavage pathway. The metabolic pathway of the upregulated *LSNL_1566-1573* is shown in Figure S5. Genes encoding certain phenol ortho-cleavage pathway enzymes were found in multiple sites on *A. lwoffii* replicons and were strongly expressed during phenol stress. Multiple copies of the gene are beneficial to microbial biodegradation by transcriptome analysis, similar to the effects of gene copy numbers on microbial biosynthesis [19]. Upregulation of the expression of benzoate hydroxylase encoded by *LSNL1569–1571* under phenol stress in *A. lwoffii* coincides with the reported inhibition of phenol degradation [20]. The downregulated genes were more abundant than the upregulated genes under phenol stress. Of the 435 downregulated genes, 255 were assigned to KEGG Orthology (KO) functional categories and matched to KEGG pathways (Table S4). These pathways, including the five additional KO categories, are shown in Figure 6. The gene expression of ATP synthase and NADH-quinone oxidoreductase in oxidative phosphorylation was significantly downregulated in the phenol-containing medium. Expression levels of ATP-binding cassette (ABC) transporters for substrates, such as urea, phosphate, sulfonate, and benzoate, were also suppressed. For example, the downregulated *LSNL_0083-0085* (KO: K11960-K11962) were annotated to encode urea transporters, which might be impacted indirectly by phenol stress effects on ABC transport. Meanwhile, the expression of genes involved in sulfite reduction from dimethyl sulfone or alkanesulfonate in sulfur metabolism was downregulated under phenol stress. Moreover, expression levels of genes related to L-lactate dehydrogenase (*LNSL_3334*), alcohol dehydrogenases (*LNSL_2862* and *LNSL_3386*), and acetate formation (Phosphate acetyltransferase *LNSL_0413*, acetate kinase *LNSL_0414*, aldehyde dehydrogenase *LNSL_3311*) were downregulated during pyruvate metabolism. Other downregulated metabolic pathways under phenol stress included phenylalanine metabolism, amino and nucleotide sugar metabolism, and fatty acid metabolism. In addition to the metabolic subsystem, cellular processes, such as quorum sensing, bacterial secretion, ribosomes, and the two-component system, were affected by phenol stress.

### 2.4. Mechanism of Adaptation to Phenol Stress in A. lwoffii NL115

**Figure 7:**
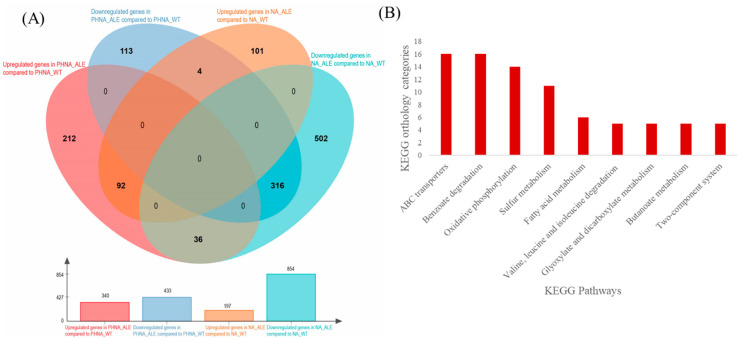
DEGs in *Acinetobacter lwoffii* NL115 in response to phenol stress. (**A**) Venn diagrams for RNA-seq of the two strains on phenol-containing medium and NaAc medium. PHNA_ALE and PHNA_WT: *A. lwoffii* NL115 and *A. lwoffii* NL1 were cultivated on the medium containing 1.3 g/L NaAc and 0.5 g/L phenol, respectively. NA_ALE and NA_WT: *A. lwoffii* NL115 and *A. lwoffii* NL1 were cultivated on 1.3 g/L NaAc medium. (**B**) Pathways enriched in *Acinetobacter lwoffii* NL115 in response to phenol stress. The ordinate corresponds to the number of KO categories in each metabolic pathway.

To explore the mechanism of adaptation to high phenol concentrations during evolution, the DEGs between *A. lwoffii* NL115 and NL1 were analyzed. Compared to levels in the starting strain NL1, 197 and 854 genes were upregulated and downregulated, respectively, in *A. lwoffii* NL115 on NaAc medium, whereas 340 upregulated and 433 downregulated genes were identified when the bacteria were cultured on phenol-containing medium (Figure 7A). Excluding the DEGs identified in the NaAc medium, 248 upregulated and 117 downregulated genes in *A. lwoffii* NL115 were determined to respond to phenol stress (Table S5). Among them, 154 upregulated and 50 downregulated genes were assigned to 145 and 49 KO function categories, respectively. Pathways that included more than five KO categories were defined as upregulated or downregulated. The absence of downregulated pathways indicated that the downregulated genes were functionally dispersed. The pathways upregulated in the evolutionary strains are shown in Figure 7B. Some transporters involved in ABC and MFS transport were upregulated in *A. lwoffii* NL115, and these proteins can transport nutrients (urea, sulfate, ammonia), metal ions (ferrous iron, mercuric ion), oligopeptides (glutathione), organic acids (cis, cis-muconate, tartrate, malonate), choline, and biopolymers. Benzoate degradation gene expression in *A. lwoffii* NL115 was significantly higher than that in *A. lwoffii* NL1, which reflects the improved positive degradation observed in the evolutionary process. The upregulation of oxidative phosphorylation indicated more ATP and carbon flux in central carbon metabolism, which is consistent with the faster growth rates of *A. lwoffii* NL115 under the same culture conditions. Sulfur metabolism and acetyl-CoA conversion in alternative carbon metabolism were generally elevated in *A. lwoffii* NL115, which might be related to CoA being conducive to phenol stress resistance [21]. In addition, fatty acid metabolism, a two-component system of nitrogen and short fatty acids, and chemotaxis were enriched in *A. lwoffii* NL115 cells. Notably, the most highly upregulated genes (log_2_FC values > 5) in *A. lwoffii* NL115 included *LNSL_1974* (alkyl hydroperoxide reductase subunit F, 5), *LNSL_ 0160* (alkyl hydroperoxide reductase subunit C, 6.3), and *LNSL_0066* (catalase, 5.9).

**Figure 8:**
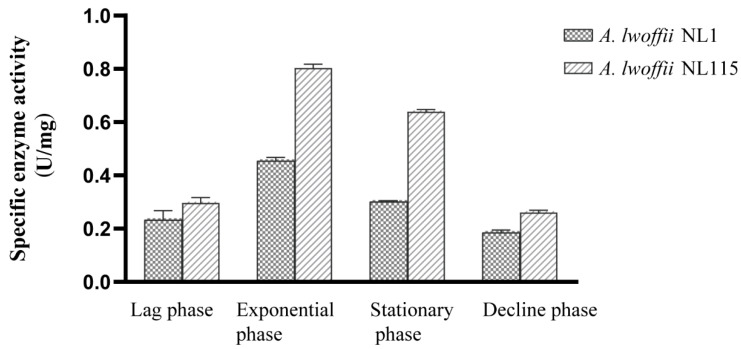
Phenol hydroxylase activity in *Acinetobacter lwoffii* NL1 and *A. lwoffii* NL115 during the entire period.

The aforementioned DEG analysis combined with genome resequencing showed that phenol hydroxylase and peroxidase in the evolutionary strain are important during the response to phenol. The crude enzyme activities of the phenol hydroxylases of *A. lwoffii* NL115 and NL1 were compared in liquid MM medium containing 0.5 g/L phenol as the sole carbon source (Figure 8). The activity of phenol hydroxylase at different growth phases varied with cell growth trends and reached its highest level at the logarithmic growth phase. The enzyme activity of *A. lwoffii* NL115 was nearly two-fold that of *A. lwoffii* NL1 in the logarithmic growth phase, which was in accordance with the log_2_FC (*A. lwoffii* NL115 relative to *A. lwoffii* NL1) in phenol hydroxylase (*LNSL_2975–2980*), which was 1.98–2.89.

**Figure 9:**
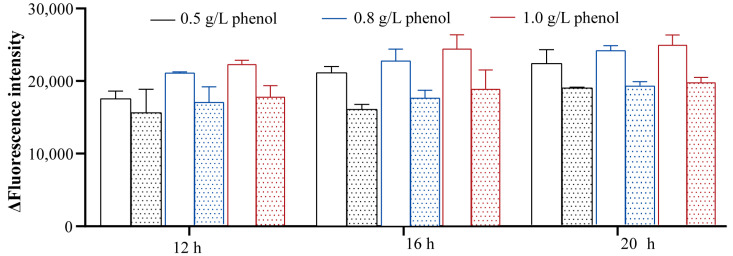
Intracellular reactive oxygen species contents under different phenol concentrations and treatment times. The blank pattern represents *A. lwoffii* NL1, and the dotted pattern represents *A. lwoffii* NL115.

The highly upregulated peroxidase activity in *A. lwoffii* NL115 could be associated with its stronger antioxidant capacity under phenol stress. Microorganisms under stress generate intracellular reactive oxygen species (ROS), and enzymatic and non-enzymatic antioxidants help them resist oxidative damage [22]. To investigate the oxidative stress caused by phenol, the ROS levels in *A. lwoffii* NL1 and NL115 were measured using the fluorescent compound DCFH-DA. As shown in Figure 9, ROS levels in *A. lwoffii* NL115 were lower than those in *A. lwoffii* NL1 for the same phenol concentration and treatment time. Moreover, the adapted strain NL115 exhibited strengthened antioxidant capacity.

**Figure 10:**
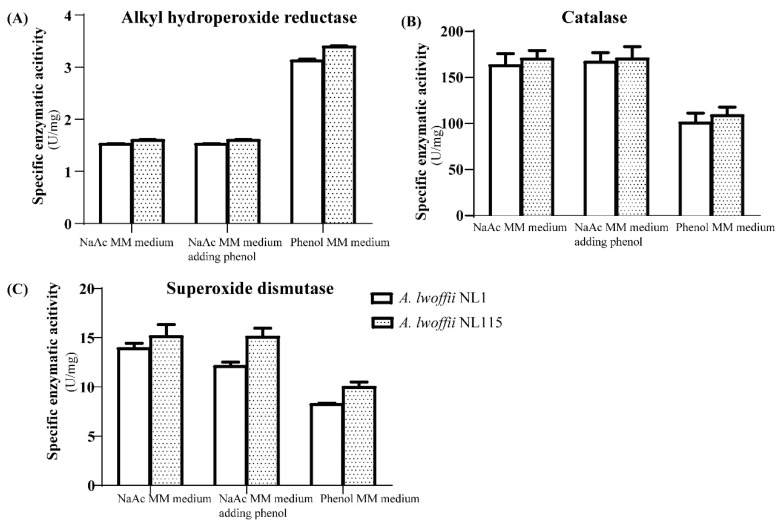
Crude enzyme activities of peroxidases from *Acinetobacter lwoffii* NL1 and *A. lwoffii* NL115. (**A**) Alkyl hydroperoxide reductase (**B**) Catalase (**C**) Superoxide dismutase. MM, minimal mineral.

Comparative transcriptomics revealed that alkyl hydroperoxide reductase and catalase levels were highly upregulated under phenol stress in *A. lwoffii* NL115. The crude enzyme activities of alkyl hydroperoxide reductase on phenol MM medium were approximately 2-fold higher than those on the two NaAc media (Figure 10A), whereas catalase activities were approximately half those on the two NaAc media (Figure 10B). Another common peroxidase superoxide dismutase showed similar activities in the two NaAc media and decreased activity in the phenol MM medium (Figure 10C). Crude enzyme activities of peroxidases thus showed differences in the three types of media, and alkyl hydroperoxide reductase played a more important role in the response to phenol stress.

**Figure 11:**
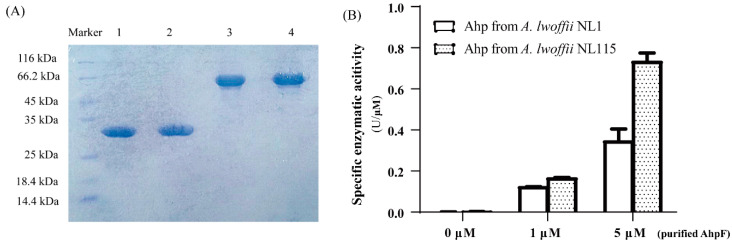
Enzyme activity of purified AhpC protein from *Acinetobacter lwoffii* NL1 and *A. lwoffii* NL115.(**A**)Purification of AhpC and AhpF proteins from BL21-LSNL1 and BL21LSNL115 via sodium dodecyl sulfate-polyacrylamide gel electrophoresis. M: protein maker; lane 1–4: protein purification results for pET-30a-*LSNL1_0160*, pET-30a-*LSNL115_0160*, pET-30a-*LSNL1_1974*, and pET-30a-*LSNL115_1974*, respectively. (**B**) Enzyme activities of alkyl hydroperoxide reductase using purified AhpC and AhpF proteins from *A. lwoffii* NL1 and NL115.

Alkyl hydroperoxide reductase (Ahp) in *A. lwoffii* was annotated as being composed of AhpC (*LNSL_1974*, subunit F) and AhpF (*LNSL_0160*, subunit C). AhpF is a common flavoprotein that transfers electrons from the reduced coenzyme I/II to the catalytic subunit AhpC. The reduced AhpC is then used to detoxify organic peroxides [23]. The crude enzyme activities of Ahp in *A. lwoffii* NL115 were slightly higher than those in *A. lwoffii* NL1, without a significant difference (Figure 10A); however, a sense mutation was found in *LNSL_1974* using comparative genomics. Therefore, the enzymatic activities of purified Ahp from *A. lwoffii* NL115 and NL1 were compared. The DNA fragments of *AhpC* and *AhpF* from *A. lwoffii* NL115 and NL1 were cloned into the plasmid pET-30a expression vector and successfully purified (Figure 11A). The NADPH-dependent peroxidase assay was carried out using tert-butyl hydroperoxide as an organic peroxide and purified AhpC and AhpF proteins from the two strains. The catalytic capacity of alkyl hydroperoxide reductase was determined to be AhpF-dependent using excess purified AhpC protein. As shown in Figure 11B, Ahp enzyme activity increased with increasing concentrations (0 μm,1 μM, and 5 μM) of purified AhpF. No enzymatic activity was observed in the absence of purified AhpF, suggesting that AhpF was required for Ahp. When adding 1 and 5 μM of the purified protein AhpF, Ahp enzyme activities from *A. lwoffii* NL115 were higher by 43.1% and 87.7% compared to those from *A. lwoffii* NL1. The activity of purified NL115-1974 was higher than that of purified NL1-1974, which enhanced alkyl hydroperoxide reductase activity in the evolutionary strain for higher phenol stress resistance.

## 3. Discussion

Phenol is the simplest phenolic and most common contaminant in polluted wastewater and sewage sludge. The study of microbial phenol degradation can serve as a reference for bioremediation of more aromatic contaminants. *A. lwoffii* NL1, with a high phenol degradation efficiency (41.67 mg/L per hour), was chosen as the starting strain in this study. The *A. lwoffii* NL115 strain, obtained via stepwise evolution, completely degraded 1.5 g/L phenol for 12 h in a shake flask and could not grow at concentrations higher than 1.5 g/L on agar plates (Figure S6). The degradation efficiency and degradation concentration were also three-fold higher than those of the starting strain, NL1. The degradation of phenol at a high concentration can rely solely on pure species such as *Burkholderia* sp. (1.5 g/L, approximately 48 h) [24], *R. opacus* PD630 (1.5 g/L, 45 h) [12], *B. brevis* (1.5 g/L, 108 h) [8], *Acinetobacter* strain V2 (1.4 g/L, approximately 24 h) [14], *C. albicans* TL3 (1.41 g/L, 50 h) [25], and *C. tropicalis* PHB5 (2.4 g/L, ~48 h) [26]. The degradation concentration of *A. lwoffii* NL115 was lower than that of *C. tropicalis* PHB5, but its degradation efficiency (125 mg/L/h) was higher. *A. lwoffii* NL115 also exhibited a high degradation capacity among these degradative bacteria and could be a promising candidate for the bioremediation of high-concentration phenol-containing environments.

Under phenol stress, activation of the β-ketoadipate and ortho-cleavage pathways was highly upregulated in *A. lwoffii*. Upregulation of the expression of genes involved in phenol degradation and utilization has also been observed in many other phenol-degrading microorganisms [12,27,28]. Metabolic processes related to cellular growth, such as oxidative phosphorylation, pyruvate metabolism, and nutrient transport, were also downregulated by phenol toxicity in bacterial cells.

**Figure 12:**
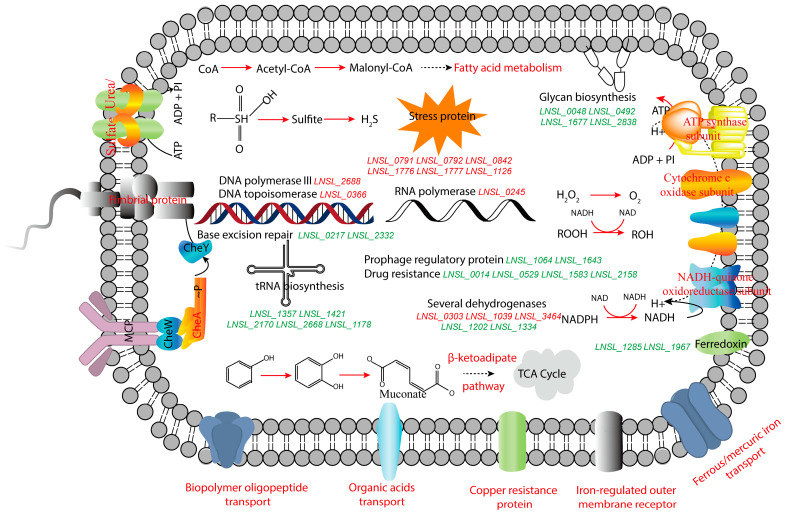
Gene expression analysis of the response to phenol stress in *Acinetobacter lwoffii* NL115. Upregulated genes and processes are shown in red, and downregulated genes and processes are shown in green. *LNSL_0048*, *LNSL_0492*, *LNSL_1677*, and *LNSL_2838* are involved in glycan biosynthesis. *LNSL_1285* and *LNSL_1967* encode ferredoxin. Among the drastically altered dehydrogenase genes were *LNSL_0303*, *LNSL_1039*, *LNSL_3464*, *LNSL_1202*, and *LNSL_1334*. *LNSL_0791*, *LNSL_0792*, *LNSL_0842*, *LNSL_1776*, *LNSL_1777*, and *LNSL_1126* encode proteins involved in stress response. In genetic information processes, *LNSL_2688*, *LNSL_0366*, *LNSL_0217*, and *LNSL_2332* were used for replication and repair, *LNSL_0245* was used for transcription, *LNSL_1357*, *LNSL_1421*, *LNSL_2170*, *LNSL_2668,* and *LNSL_1178* were used for translation, *LNSL_1064* and *LNSL_1643* were prophage regulatory proteins. *LNSL_0014*, *LNSL_0529*, *LNSL_1583*, and *LNSL_2158* were involved in drug resistance. Table S5 contains the thorough annotations of these genes.

Furthermore, a systematic comparison of gene expression between *A. lwoffii* NL1 and NL115 revealed the three main metabolic responses underlying the adaptation of *A. lwoffii* to phenol. As shown in Figure 12, (i) the direct degradation was improved. The ortho-cleavage and β-ketoadipate pathway activation was upregulated after adaptive evolution. Levels of transporters for benzoate and cis-muconate were also upregulated in NL115. Upregulation of benzoate transporters may reduce competition for their common protocatechuate branch of the β-ketoadipate pathway in phenol and benzoate metabolism. Because of intracellular intermediate efflux, upregulation of the cis, cis-muconate transporter may favor the direction of degradation of the β-ketoadipate pathway. (ii) Stress resistance was improved. The upregulated proteins in the NL115 cells were annotated in response to various stressors. Excessive ROS causes oxidative damage to microbes [29], and gene expression and enzymatic activities related to the reduction of hydrogen peroxide and hydroperoxide were enhanced in *A. lwoffii* NL115, which would have been induced to eliminate ROS and facilitate resistance to oxidative stress. The upregulated genes encoding alkanesulfonate monooxygenase, sulfate transport, sulfonate-binding proteins, and sulfite reductases suggest enhanced sulfur metabolism in *A. lwoffii* NL115. Sulfur is an important antioxidant under stress [30] that directly affects cysteine biosynthesis, providing a precursor for CoA formation. The upregulated malonyl-CoA in fatty acid metabolism, in turn, could affect the cell membrane composition of *A. lwoffii* NL115. Similarly, expression levels of several genes involved in glycan biosynthesis were downregulated in *A. lwoffii* NL115, and these are related to the cell wall composition. The cell wall and membrane might thus participate in microbial stress responses [31,32]. (iii) Vital activities could be improved. Levels of genes encoding the chemotaxis sensor kinase CheA and fimbrial proteins were upregulated, indicating stronger cell motility in *A. lwoffii* NL115. Levels of genes encoding several dehydrogenases were significantly upregulated or downregulated, indicating the frequent regeneration of redox sites as energetic electron donors. The enhanced oxidative phosphorylation revealed increased energy transfer and ATP generation in *A. lwoffii* NL115, which was in accordance with the faster cell growth of *A. lwoffii* NL115 on the same medium.

Omics analysis and biochemical phenotypes confirmed that phenol hydroxylase and alkyl hydroperoxide reductase are important for the stronger degradation capacity of *A. lwoffii* NL115. Considering the sense mutations in *LSNL_2977* and *LSNL_1974* identified through comparative genomics, the 3D structures of the two enzymes were compared between *A. lwoffii* NL115 and NL1 strains. *LSNL_2977* encodes the phenol hydroxylase protein component P2 as a reductase component that transfers electrons from NADH [33]. As shown in Figure S7, the mutation site in the loop region (position 35) between strands 2 and 3 changed from alanine to valine, with more side chain groups. The 2.67 RMSD value suggested the similarity of phenol hydroxylase protein component P2 from *A. lwoffii* and *Pseudomonas* sp. CF600 to the described structure (PDB:1HQI). Their secondary structural comparison can be seen in Figure S8. *LSNL_1974* encodes alkyl hydroperoxide reductase subunit F, which transfers electrons from NAD(P)H to AhpC for ROS detoxification [34]. The mutation site (position 371, A to T, shown in Figure S9) is located in the NADH-binding domain of the subunit AhpF [35]. When the predicted AhpF from *A. lwoffii* NL1 and NL115 were respectively compared to the known AhpF structures from *Salmonella enterica* (PDB: 1HYU) and *Escherichia coli* (PDB: 4O5Q), the RMSD values were 11.833 and 10.563 with the 1HYU, and 20.832 and 23.895 with the 4O5Q. The large RMSD values indicate that the predicted Ahp structures differed significantly from the characterized AhpF from *S. enterica* and *E. coli*.

In this study, we used adaptive laboratory evolution to develop a new strain with improved degradation ability. The resulting strain NL115 could break down 1.5 g/L phenol in 12 h. Furthermore, *A. lwoffii* NL115 outperformed the starting strain NL1 in terms of phenol degradation and tolerance. Comparative genomics and transcriptomics performed to investigate the molecular adaptation in *A. lwoffii* NL115 revealed degradative metabolism, stress resistance, and growth-associated processes in A. lwoffii adapted to phenol stress. Furthermore, phenol hydroxylase and alkyl hydroperoxide reductase were identified as important enzymatic activities that contributed to phenol response in *A. lwoffii* NL115. Overall, *A. lwoffii* NL115 appears to be a potential choice for the practical remediation of phenol-polluted settings, and the mechanism behind efficient phenol degradation could guide advancements in phenol degradation by other bacteria.

## 4. Material and Methods

### 4.1. Strains and Media

The starting strain was the previously isolated phenol-degrading *A. lwoffii* NL1 (CCTCC NO: M2014329). The evolved strain *A. lwoffii* NL115 was obtained through ALE. *Escherichia coli* DH5α and BL21 strains were purchased from Invitrogen.

Luria–Bertani (LB) medium was used to activate the cultures. Minimal mineral (MM) media (NH_4_Cl, 1.0 g/L; NaH_2_PO_4_, 1.0 g/L; K_2_HPO_4_, 3.0 g/L; KCl, 0.15 g/L; MgSO_4_·7H_2_O, 0.3 g/L; CaCl_2_, 0.01 g/L; FeSO_4_·7H_2_O, 2.5 mg/L; pH 7.0) containing different concentrations of phenol were used for ALE and phenol tolerance test (adding 1.5% agar). Liquid MM medium containing 1.3 g/L Sodium acetate (NaAc) as a carbon source was used for transcriptomic analysis with the addition of 0, 0.5, and 1.5 g/L phenol. Liquid MM medium containing 0.5 g/L phenol as a carbon source was used to compare the phenol degradation abilities and tolerance levels of *A. lwoffii* NL1 and NL115.

### 4.2. Adaptive Evolution Process (ALE)

ALE was performed in 50 mL of shake culture (10% *v*:*v* inoculum amount) at 28 °C and 200 rpm. The initial phenol stress was 0.5 g/L, and the stress concentration of phenol was increased by 10–50 mg/L in every cycle. *A. lwoffii* NL1 in LB test tubes after overnight culture was diluted to achieve an optical density (OD)_600_ of 0.5 and then inoculated into liquid MM medium with phenol as the sole carbon source. The OD_600_ of the biomass was measured when the phenol was completely degraded. The bacterial fluid was again adjusted to an OD_600_ of 0.5 and inoculated into fresh liquid MM medium with higher concentrations of phenol.

The evolution process was repeated until bacterial cultures were obtained under 1.5 g/L phenol stress. Bacterial cultures were appropriately diluted and coated onto MM plates (1.5% agar) containing 1.5 g/L phenol. The colony-forming isolates were further purified via cultivation on the same plates for 3 days. The resulting single colony was inoculated into liquid MM medium containing 1.5 g/L phenol and preserved as *A. lwoffii* NL115 after the phenol was completely degraded.

### 4.3. Phenotypic Comparison of A. lwoffii NL1 and NL115

Colony-forming isolates of *A. lwoffii* NL1 and NL115 on the LB plates were inoculated in LB test tubes. At their logarithmic growth phase, cells were collected and resuspended in sterile phosphate buffer solution (PBS) to achieve an OD_600_ of 0.5. The diluted bacterial suspension was used for phenotypic comparison of *A. lwoffii* NL1 and NL115.

To compare the trends in cell growth and phenol degradation, the washed bacterial suspension was cultivated in 50 mL of liquid MM medium (10% *v*/*v* inoculum) containing 0.5 g/L phenol. Liquid cultures, at 28 °C and 200 rpm, were taken every 1–2 h to determine the OD_600_ of the biomass and supernatants of the cultures were treated for measuring the OD_510_ of phenol using the 4-aminoantipyrine colorimetric method [36].

The growth of *A. lwoffii* NL115 on phenol-containing plates was investigated. The washed bacterial suspensions were diluted based on a gradient of six concentrations, specifically 10^−1^, 10^−2^, 10^−3^, 10^−4^, 10^−5^, and 10^−6^, and spotted onto MM agar plates containing phenol at concentrations ranging from 0 to 1.6 g/L. The growth phenotype of *A. lwoffii* NL115 on the phenol plate was observed after 2–3 days. The growth of *A. lwoffii* NL115 on MM agar plates using sodium acetate as the carbon source was investigated. Following a gradient of five concentrations—10^−1^, 10^−2^, 10^−3^, 10^−4^, and 10^−5^—the washed bacterial suspensions were diluted and spotted onto MM agar plates with different concentrations of sodium acetate. The C molar masses of sodium acetate and phenol were the same at every concentration. After two to three days, the growth phenotype of *A. lwoffii* NL115 on the MM agar plates was seen.

Following acridine orange (AO) and ethidium bromide (EB) staining, the survival of cells treated with phenol was qualitatively assessed using fluorescence microscopy. The washed bacterial suspensions of *A. lwoffii* NL1 and NL115 were treated for 2 h at 28 °C with shaking at 50 rpm in the presence of 0.5 g/L and 1.0 g/L phenol. The cells were washed with PBS to remove the residual phenol and fluorescently stained with AO/EB for 10 min. Cell survival was observed based on fluorescence microscopy with the green and red fluorescence channels [37]. 

The survival of cells treated with phenol after acridine orange (AO) staining was evaluated using a microplate reader to measure fluorescence intensity. *A. lwoffii* NL1 and NL115 washed bacterial samples were treated for 4 h at 28 °C and 50 rpm in the presence or absence of 0.5 g/L and 1.0 g/L phenol. After washing the cells with PBS to remove the leftover phenol, they were fluorescently stained with AO for 10 min at 37 °C. The dye solution was extracted from the cells as much as possible by centrifugation (3 min at 800 rpm). The cells were resuspended in PBS, and the fluorescence intensity of AO was measured at excitation and emission wavelengths of 488 and 530 nm, respectively.

Intracellular reactive oxygen species (ROS) were measured on a microplate reader using 2′,7′-dichlorofluorescein diacetate (DCFH-DA). The bacterial suspensions (OD_600_ = 0.5) of *A. lwoffii* NL1 and NL115 were incubated on liquid MM medium with different concentrations of phenol (0.5, 0.8, and 1.0 g/L) as the sole carbon source. Cultures were collected at different cultivation times (12, 16, and 18 h) and diluted to an OD_600_ of 0.1 with sterile PBS buffer. The bacterial suspensions were resuspended using 10 µM DCFH-DA and incubated at 37 °C in the dark for 30 min. The cells were then washed three times with sterile PBS buffer. The fluorescence intensity of ROS was measured at excitation and emission wavelengths of 488 and 525 nm, respectively.

### 4.4. Activity of Crude Enzymes

Cells of *A. lwoffii* NL1 and NL115 at certain growth phases were collected, washed, and resuspended in 0.1 M K_3_PO_4_ buffer. Cells were lysed to near clarity for 15 min (4 s ON, 8 s OFF) in an ultrasonic cell crusher. The supernatants were used as crude enzymes, and the total protein concentration was determined using the Bradford method [38]. Phenol hydroxylase activity [39] was measured as the change in absorbance (A) at 340 nm in a reaction mixture containing 0.2 mM FAD, 0.1 mM NADPH, 1 mM phenol, 0.1 M K_3_PO_4_ buffer (pH 7.5), and crude enzymes. Catalase activity [40] was determined based on the change in absorbance (A) at 240 nm in a reaction mixture containing 20 mM H_2_O_2_, 50 mM K_3_PO_4_ buffer (pH 7.0), and the crude enzymes. Alkyl hydroperoxide reductase activity [41] was determined based on the change in the absorbance of xylenol orange at 560 nm. The Fox reagent contained 125 μM xylenol orange, 250 μM ammonium iron (II) sulfate, 100 μM sorbitol, and 25 mM H_2_SO_4_ and was added to the reaction mixtures of crude enzymes and 10 mM H_2_O_2_.

### 4.5. Genome Resequencing and Analysis

High-quality genomic DNA of *A*. *lwoffii* NL115 was analyzed using a Qubit fluorometer. Raw reads generated on the DNBSEQ platform (BGI, Shenzhen, China) were assembled into contigs and scaffolds using SOAPdenovo software v1.05 [42]. For comparative genomic analysis, the initial single nucleotide polymorphisms were detected using MUMmer software [43] and filtered using the BLAT program version 34 [44]. The initial insertion–deletion mutations (indels) were checked using the LASTZ program [45] and filtrated using the BLAT program version 34 [44].

### 4.6. RNA Sequencing and Analysis

*A. lwoffii* NL1 and NL115 were cultured in three different media for RNA-Seq during mid-exponential growth. The two strains were collected at 4 h on 1.3 g/L NaAc as the carbon source and designated as NA_WT and NA_ALE. The two strains at 5 h on 1.3 g/L NaAc, after adding 0.5 g/L phenol as a carbon source, were collected and designated as PHNA_WT and PHNA_ALE, respectively. *A. lwoffii* NL115 at 9 h on 1.3 g/L NaAc, with the addition of 1.5 g/L phenol as the carbon source, was collected and designated as 15PHNA_ALE. Three biological samples of each treatment were submitted to a sequencing company (Shanghai Majorbio Bio-Pharm Technology Co., Ltd., Shanghai, China) to complete the RNA-seq. Total RNA was isolated from the five samples using chloroform, ethanol, and a Bacteria Total RNA Isolation Kit (Sangon Biotech, Shanghai, China). Total RNA was checked for purity and integrity prior to cDNA library construction. The qualified libraries were sequenced as 150–200 bp paired-end reads on the Illumina HiseqTM 4000 platform. Clean reads were mapped to the *A. lwoffii* NL1 genome using Bowtie software [46]. The transcripts per kilobase of the exon model per million mapped reads (TMP) were calculated to represent gene expression using RSEM software [47]. DEGs (fold-change (FC) > 2 and *p*-adjusted < 0.05) between groups were identified using the DESeq R package [48]. The KO functional categories were used to integrate DEGs into the KEGG pathway. All the related KO categories were mapped to the KEGG pathway using the Web server KEGG Mapper (www.kegg.jp/kegg/mapper/, 14 September 2023). The pathways containing more than five KO categories were thought to be functionally enriched.

### 4.7. Transcriptional Analysis Using Quantitative Real-Time PCR (qRT-PCR)

Qualified RNA was used to synthesize cDNA using the HiScript^®^ III RT SuperMix. The cDNA templates were subjected to PCR using primer pairs (qRT-PCR primers are listed in Table S3) and were then used to quantify gene expression on a CFX Connect Real-Time PCR Detection System (Bio-Rad, Hercules, CA, USA). The 20 µL mixture for qRT-PCR contained 10 µL of ChamQ Universal SYBR qPCR Master Mix (2×), 0.4 µL of each forward and reverse primer, 2 µL of cDNA templates (50 ng/μL), and 7.2 µL of double-distilled H_2_O. The relative expression levels were determined using the 2^−ΔΔCt^ method [49] with 16S rRNA genes as the reference. The experiments were performed with three biological replications. The log2 transformed fold changes acquired by RNA-seq and qRT-PCR were plotted as a scatter plot to assess the consistency of the two methods’ results.

### 4.8. Enzymatic Activity of Purified Alkyl Hydroperoxide Reductase

Alkyl hydroperoxide reductases from *A*. *lwoffii* NL1 and NL115 were expressed using the vector pET30a in *E. coli* BL21 and purified using a nickel-affinity chromatography column. Genomic DNA of *A*. *lwoffii* NL1 and NL115 was extracted as a template to amplify the F and C subunits of *ahp* genes using the *AhpF*-F/R (5′ATCGGATCCGAATTCATGTTAGATCAAAATACTTCAGCCC3′ and 5′ GTGGTGGTGCTCGAGTTATTGCCCAGAACGGATGA3′. Restriction enzyme sites were underlined.) and *AhpC*-F/R (5′ ATCGGATCCGAATTCATGAGTTTAATCAATACTGAAATC3′ and 5′ GTGGTGGTGCTCGAGTTAGATTTTACCCACCAGGTC3′. Restriction enzyme sites were underlined.) primer pairs, respectively. The four target fragments were identified via agarose gel electrophoresis (1%) and recovered using the MiniBEST Agarose Gel DNA Extraction Kit (TaKaRa, Maebashi, Japan). Ligation of the four fragments with the vector pET-30a (*Eco*RI and *Xho*I digestion) yielded pET-30a-*LSNL_1974*, pET-30a-*LSNL115_1974*, pET-30a-*LSNL_0160*, and pET-30a-*LSNL115_0160*. The recombinant plasmids were transformed into *E. coli* BL21 cells via heat shock. Positive transformants were cultivated to logarithmic growth phase in 100 mL of LB liquid medium at 37 °C, and 0.7 mM isopropylthio-β-D-galactoside was added to the cultures for protein expression (at 160 rpm, 28 °C for 4 h). After sonication lysis, as described in the above extraction of crude enzymes, the supernatants of the harvested cell suspension were collected via centrifugation (12,000× *g*, 4 °C for 30 min) for protein purification. The supernatants were fully and slowly bound to the HisPur™ (Dhule, India) Ni-NTA resins at 4 °C for 2 h. The resins were repeatedly washed with 20 mM imidazole to remove nonspecifically bound proteins. His-tagged fusion proteins were eluted using 250 mM imidazole and analyzed via sodium dodecyl sulfate-polyacrylamide gel electrophoresis. Purified Ahp proteins from *A*. *lwoffii* NL1 (LSNL_1974 and LSNL_0160) and NL115 (LSNL115_1974 and LSNL115_0160) were concentrated using dialysis and ultrafiltration.

Hydroperoxidase activity of the purified AhpC protein was measured as the decrease in absorbance at 340 nm [50]. Ahp proteins (15 μM purified AhpC and 0–5 μM purified AhpF) from *A*. *lwoffii* NL1 and NL115 were mixed with 250 μM NADPH in HEPES buffer (pH7.5) at 30 °C for 3 min. Enzymatic reactions were initiated by adding a hydroperoxide substrate (1.5 mM tert-butyl hydroperoxide), and the absorbance at 340 nm was immediately read every 20 s.

## Data Availability

RNA-seq data of *A. lwoffii* has been deposited to NCBI (PRJNA999462). Other data and materials are available from the corresponding author.

## Supplementary Materials

The following supporting information can be downloaded at https://www.mdpi.com/article/10.3390/ijms242216529/s1 Table S1. Summary of sense mutations of genes in *A. lwoffii* NL115; Table S2. Genes used for qRT-PCR; Table S3. Primers used for qRT-PCR analysis; Table S4. Summary of the differentially expressed genes shared by *A. lwoffii* NL1 and NL115 in the presence of phenol; Table S5. Summary of the differentially expressed genes in *A. lwoffii* NL115 compared to *A. lwoffii* NL1 in phenol presence; Figure S1. Pot assays of *Acinetobacter lwoffii* NL115 growth on sodium acetate plates with different concentrations; Figure S2. A boxplot for the CT values of candidate genes from RT-qPCR analysis; Figure S3. The melting curves of the qRT-PCR products; Figure S4. Accuracy of RNA-seq results verified through qRT-PCR; Figure S5. Metabolic pathway of the upregulated gene cluster *LSNL_1566-1573*; Figure S6. *Acinetobacter lwoffii* NL1 and NL115 grown in minimal mineral medium using more than 1.5 g/L phenol as the sole carbon source; Figure S7.Predicted 3D structures of phenol hydroxylase protein component P2; Figure S8. Predicted 3D structures of phenol hydroxylase protein component P2 aligned to the characterized PDB structure (1HQI); Figure S9. Predicted 3D structure of AhpF.
