# Supplementary material for: Comparative Genomic and Transcriptomic Analysis of Phenol Degradation and Tolerance in Acinetobacter lwoffii through Adaptive Evolution
Source: Int J Mol Sci. 2023 Nov 20;24(22):16529. doi: 10.3390/ijms242216529 (PMC10671910; doi:10.3390/ijms242216529)
Supplement: Supplementary file 1 [file ijms-24-16529-s001.zip › Figure S1.pdf]

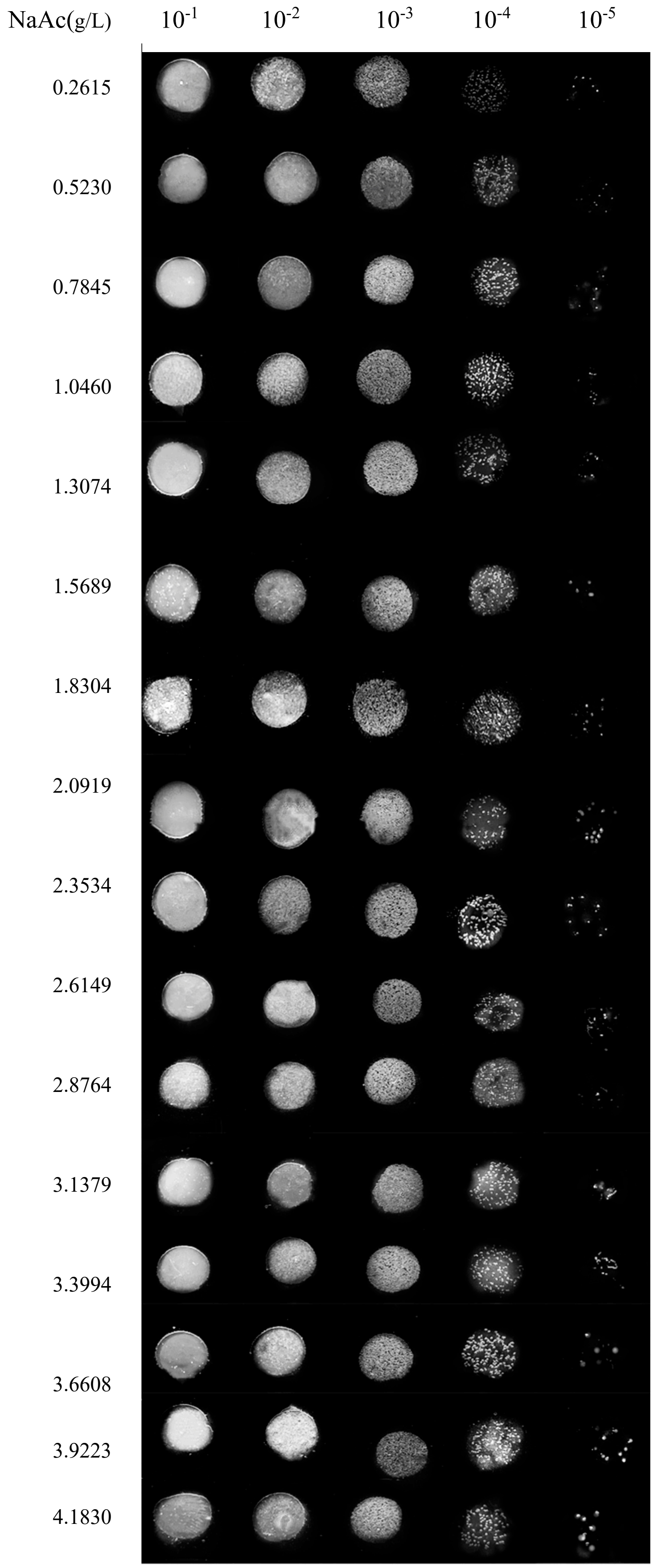

Figure S1. Pot assays of *Acinetobacter lwoffii* NL115 growth on sodium acetate

plates with different concentrations.
