# Supplementary material for: Comparative Genomic and Transcriptomic Analysis of Phenol Degradation and Tolerance in Acinetobacter lwoffii through Adaptive Evolution
Source: Int J Mol Sci. 2023 Nov 20;24(22):16529. doi: 10.3390/ijms242216529 (PMC10671910; doi:10.3390/ijms242216529)
Supplement: Supplementary file 1 [file ijms-24-16529-s001.zip › Figure S7.pdf]

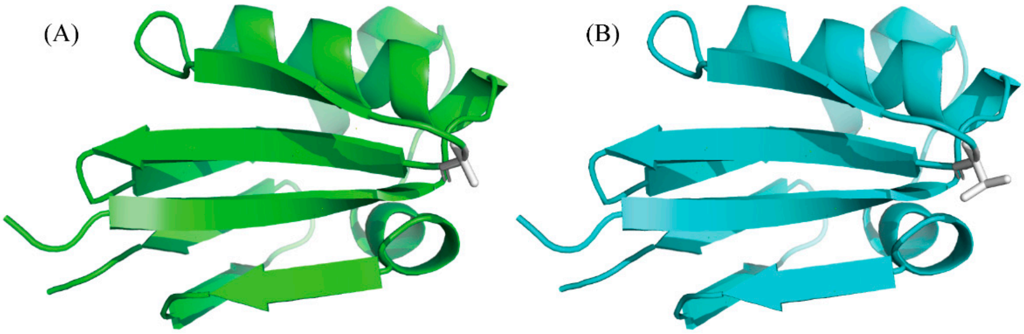

Figure S7. Predicted 3D structures of phenol hydroxylase protein component P2.

(A) in LSNL_2977 of *Acinetobacter lwoffii* NL1 and (B) in LSNL_2977 of *A. lwoffii* NL115. The stick molecules in white denote alanine (A) and valine (B) at position 35.
