# Supplementary material for: Comparative Genomic and Transcriptomic Analysis of Phenol Degradation and Tolerance in Acinetobacter lwoffii through Adaptive Evolution
Source: Int J Mol Sci. 2023 Nov 20;24(22):16529. doi: 10.3390/ijms242216529 (PMC10671910; doi:10.3390/ijms242216529)
Supplement: Supplementary file 1 [file ijms-24-16529-s001.zip › Figure S8.pdf]

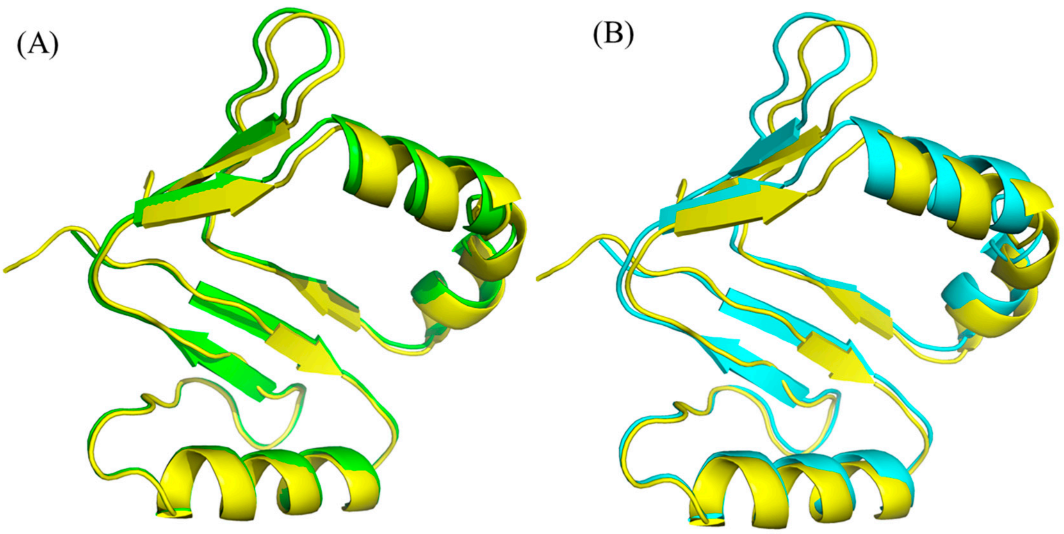

Figure S8. Predicted 3D structures of phenol hydroxylase protein component P2 aligned to the characterized PDB structure (1HQI)

(A) LSNL_2977 of *Acinetobacter lwoffii* NL1 and (B) LSNL_2977 of *A. lwoffii* NL115. The PDB structure 1HQI is highlighted in yellow.
