# Supplementary material for: Comparative Genomic and Transcriptomic Analysis of Phenol Degradation and Tolerance in Acinetobacter lwoffii through Adaptive Evolution
Source: Int J Mol Sci. 2023 Nov 20;24(22):16529. doi: 10.3390/ijms242216529 (PMC10671910; doi:10.3390/ijms242216529)
Supplement: Supplementary file 1 [file ijms-24-16529-s001.zip › Table S2.pdf]

Table S2. Genes used for qRT-PCR

Gene	Product	Location
*LNSL*_0414	Acetate kinase	chromosome
*LNSL*_1568	Catechol 1,2-dioxygenase	chromosome
*LNSL*_1583	HTH-type transcriptional regulator QacR	chromosome
*LNSL*_1838	Alkyl hydroperoxide reductase subunit C	chromosome
*LSNL*_1842	alkyl hydroperoxide reductase subunit F	chromosome
*LNSL*_1900	Superoxide dismutase	chromosome
*LNSL*_1927	Hypothetical protein	chromosome
*LSNL*_1974	Alkyl hydroperoxide reductase subunit F	chromosome
*LNSL*_2975	Phenol hydroxylase P0 protein	megaplasmid pNL1
*LNSL*_2976	Phenol hydroxylase P1 protein	megaplasmid pNL1
LNSL_2980	Phenol hydroxylase P5 protein	megaplasmid pNL1
LNSL_2981	Catechol 1,2-dioxygenase	megaplasmid pNL1
*LNSL*_3000	3-oxoadipate enol-lactonase	megaplasmid pNL1
*LNSL*_0302	Hypothetical protein	megaplasmid pNL1
*LNSL*_3005	Muconate cycloisomerase	megaplasmid pNL1
