# Supplementary material for: Comparative Genomic and Transcriptomic Analysis of Phenol Degradation and Tolerance in Acinetobacter lwoffii through Adaptive Evolution
Source: Int J Mol Sci. 2023 Nov 20;24(22):16529. doi: 10.3390/ijms242216529 (PMC10671910; doi:10.3390/ijms242216529)
Supplement: Supplementary file 1 [file ijms-24-16529-s001.zip › Table S3.pdf]

Table S3. Primers used for qRT-PCR analysis

Primer	Sequences (5′ to 3′)	amplicon size	PCR efficiency (E)
qRT-16S rRNA-F	CTCGCAGAATAAGCACCG	177	101%
qRT-16S rRNA-R	CTCCCATACTCTAGCCAACC		
qRT-*LSNL*_1842-F	CCAGGACGAAGTAGAAGAAC	150	103%
qRT-*LSNL*_1842-R	CAATCACCAGGACATCAAAG		
qRT-*LNSL*_1568-F	GATGCCGGTCAAGCCAATG	169	104%
qRT-*LNSL*_1568-R	AGCCGACAGACTCAGGAGC		
qRT-*LNSL*_1974-F	TCGCTAAAGTCACTTCCAC	148	108%
qRT-*LNSL*_1974-R	AAGACCCAAGGTCCTAAAT		
qRT-*LNSL*_1583-F	TGAAGAGCGTTTAACCAA	129	104%
qRT-*LNSL*_1583-R	GCACTTTCTAAAGCCACC		
qRT-*LNSL*_1927-F	TCGGCGACCATGTTTTATTCAT	123	107%
qRT-*LNSL*_1927-R	ACTCAAACCAATCAGCGCC		
qRT-*LNSL*_0414-F	AATGGTAGCTCGACCTGTGC	139	105%
qRT-*LNSL*_0414-R	GGTTTTTCGCCAGGAAGCTG		
qRT- *LNSL*_3000-F	GCTGCATCACGCTGGTTTAC	122	104%
qRT- *LNSL*_3000-R	GCCAAAGCTTCACAGCAACT		
qRT- *LNSL*_2975-F	TTGATGCCGCATTTGTCGAG	103	103%
qRT- *LNSL*_2975-R	TTGGTGGCACAAAACTCACG		
qRT- *LNSL*_2976-F	AACACGGGCGAATCTTTA	198	108%
qRT- *LNSL*_2976-R	CTGATGCTGTGCCAACCA		
qRT- *LNSL*_1900-F	CCCGAGCAACTCAAACAG	150	103%
qRT- *LNSL*_1900-R	TGCCTCGAATCCTCCTAA		
qRT- *LNSL*_3005-F	TTCGCAGCCCTCAAGTAA	102	104%
qRT- *LNSL*_3005-R	CTCATGGGTCCACAAAGT		
qRT- *LNSL*_1838-F	CCGACTGAACTTGGTGAC	121	103%
qRT- *LNSL*_1838-R	CTTCTGAAGAATCGTGCC		
qRT- *LNSL*_0302-F	GTTCTACCTAATCTGGCG	123	105%

qRT- *LNSL*_0302-R	ACGTAGTGACTTGCGAAT		
qRT- *LNSL*_2980-F	TTTCAGGCAGGGCAGTA	299	101%
qRT- *LNSL*_2980-R	CCCGATTCAAGCAGGTC		
qRT- *LNSL*_2981-F	TAACCGCCCATCTCACG	121	105%
qRT- *LNSL*_2981-R	TTCACGGGTAGCAAAGG		
