# Supplementary figures and images for: Comparative Genomic and Transcriptomic Analysis of Phenol Degradation and Tolerance in Acinetobacter lwoffii through Adaptive Evolution
Source: Int J Mol Sci. 2023 Nov 20;24(22):16529. doi: 10.3390/ijms242216529 (PMC10671910; doi:10.3390/ijms242216529)

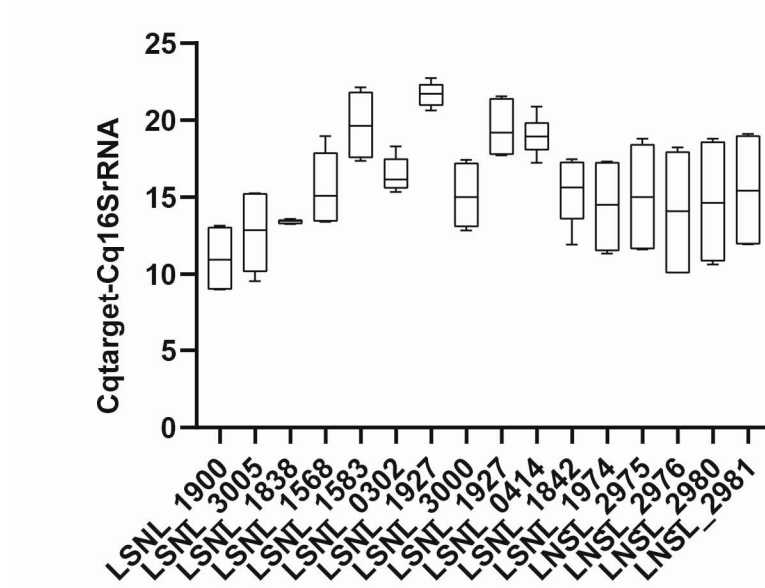

**Figure S2. A boxplot for the CT values of candidate genes from RT-qPCR analysis.**

Supplement: Supplementary file 1 [file ijms-24-16529-s001.zip › Figure S2.pdf]

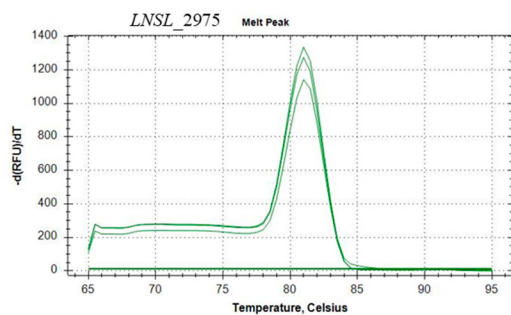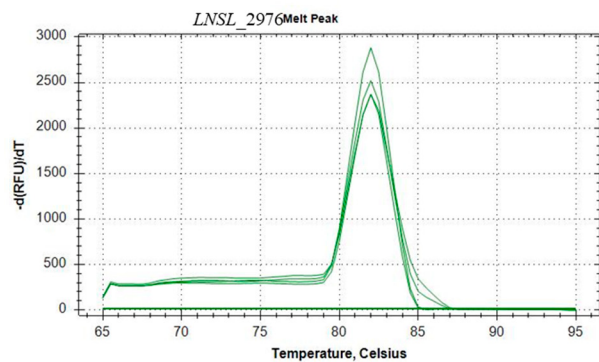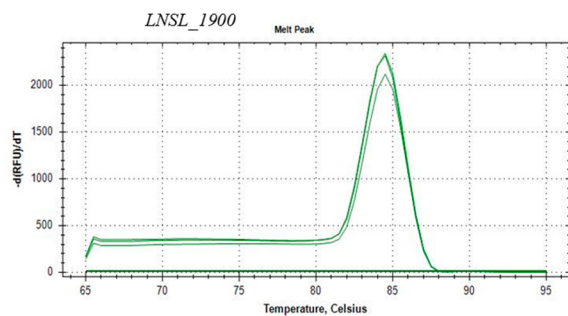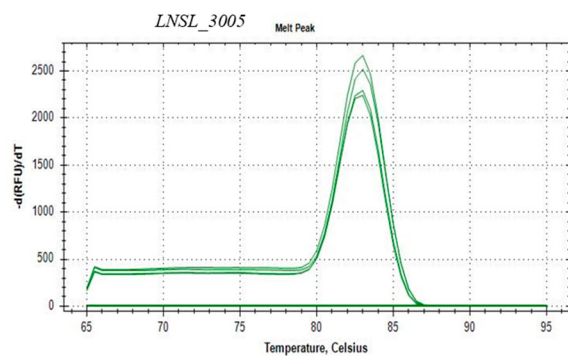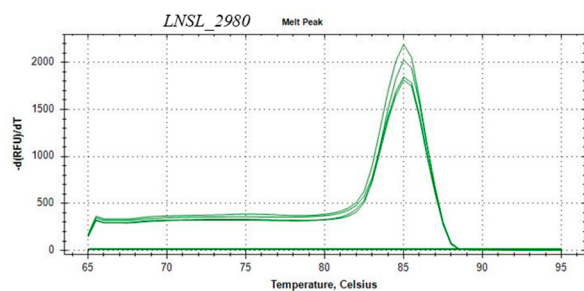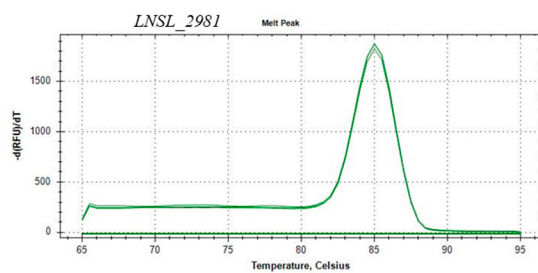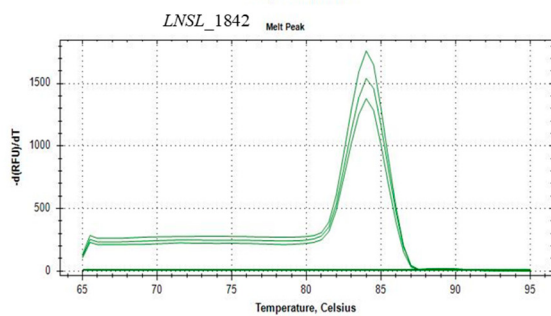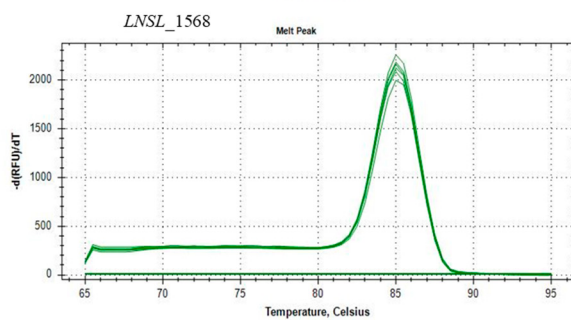

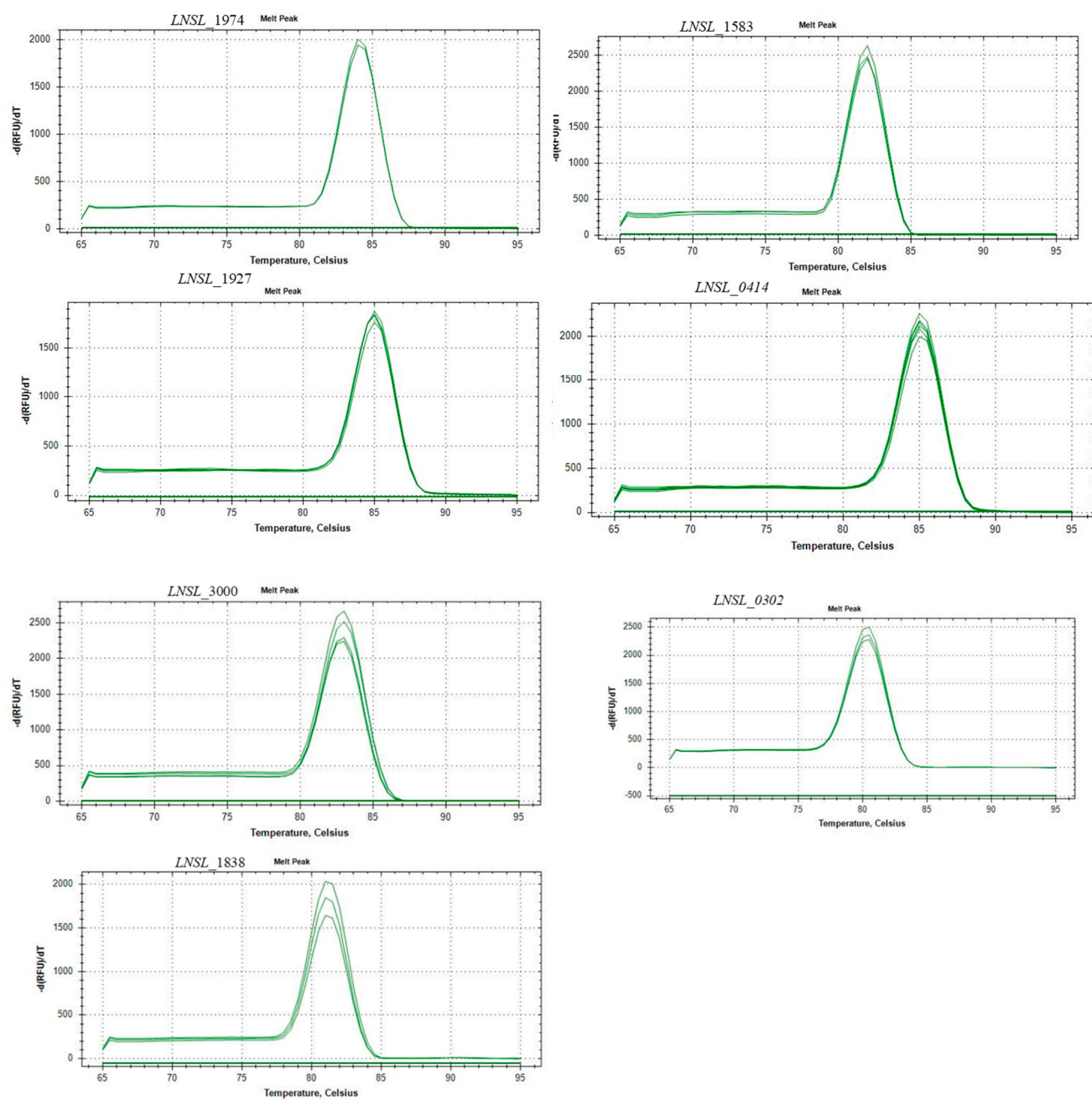

**Figure S3. The melting curves of the qRT-PCR products.**

Supplement: Supplementary file 1 [file ijms-24-16529-s001.zip › Figure S3.pdf]

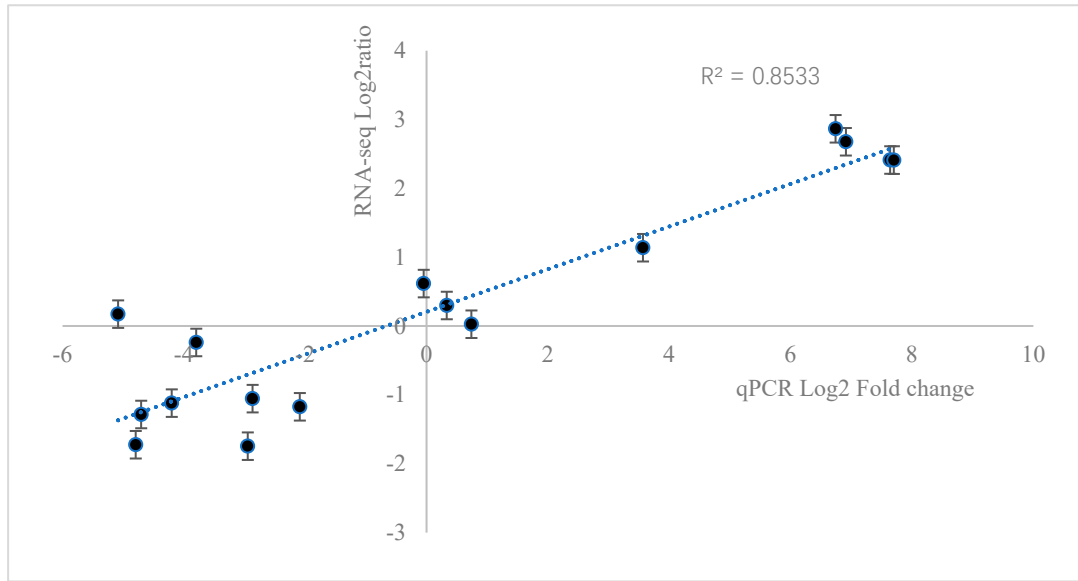

**Figure S4. Accuracy of RNA-seq results verified through qRT-PCR.**

Supplement: Supplementary file 1 [file ijms-24-16529-s001.zip › Figure S4.pdf]

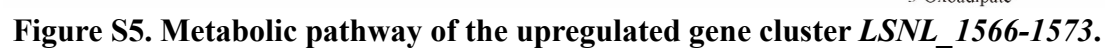

**Figure S5. Metabolic pathway of the upregulated gene cluster *LSNL\_1566-1573*.**

Supplement: Supplementary file 1 [file ijms-24-16529-s001.zip › Figure S5.pdf]
